# Inappropriate antibiotic prescribing and its determinants among outpatient children in 3 low- and middle-income countries: A multicentric community-based cohort study

**DOI:** 10.1371/journal.pmed.1004211

**Published:** 2023-06-06

**Authors:** Antoine Ardillon, Lison Ramblière, Elsa Kermorvant-Duchemin, Touch Sok, Andrianirina Zafitsara Zo, Jean-Baptiste Diouf, Pring Long, Siyin Lach, Fatoumata Diene Sarr, Laurence Borand, Felix Cheysson, Jean-Marc Collard, Perlinot Herindrainy, Agathe de Lauzanne, Muriel Vray, Elisabeth Delarocque-Astagneau, Didier Guillemot, Bich-Tram Huynh, On behalf of the BIRDY study group

**Affiliations:** 1 Université Paris-Saclay, UVSQ, Inserm, CESP, Anti-infective evasion and pharmacoepidemiology team, Montigny-Le-Bretonneux, France; 2 Institut Pasteur, Université Paris Cité, Epidemiology and Modelling of Antibiotic Evasion (EMAE), Paris, France; 3 AP-HP, Hôpital Necker-Enfants malades, Department of Neonatal medicine, Université Paris Cité, Paris, France; 4 Ministry of Health, Phnom Penh, Cambodia; 5 Peadiatric Ward, Centre Hospitalier de Soavinandriana, Antananarivo, Madagascar; 6 Centre Hospitalier Roi Baudouin Guédiawaye, Dakar, Sénégal; 7 Epidemiology and Public Health Unit, Institut Pasteur du Cambodge, Phnom Penh, Cambodia; 8 Center for Tuberculosis Research, Division of Infectious Diseases, Johns Hopkins University School of Medicine, Baltimore, Maryland, United States of America; 9 Sorbonne Université, UMR CNRS 8001, LPSM, Paris, France; 10 Experimental Bacteriology Unit, Institut Pasteur de Madagascar, Antananarivo, Madagascar; 11 Epidemiology Unit, Institut Pasteur de Madagascar, Antananarivo, Madagascar; 12 Epidemiology of Infectious Diseases Unit, Institut Pasteur de Dakar, Dakar, Sénégal; 13 AP-HP. Paris Saclay, Public Health, Medical Information, Clinical research, Le Kremlin-Bicêtre, France

## Abstract

**Background:**

Antibiotic resistance is a global public health issue, particularly in low- and middle-income countries (LMICs), where antibiotics required to treat resistant infections are not affordable. LMICs also bear a disproportionately high burden of bacterial diseases, particularly among children, and resistance jeopardizes progress made in these areas. Although outpatient antibiotic use is a major driver of antibiotic resistance, data on inappropriate antibiotic prescribing in LMICs are scarce at the community level, where the majority of prescribing occurs. Here, we aimed to characterize inappropriate antibiotic prescribing among young outpatient children and to identify its determinants in 3 LMICs.

**Methods and findings:**

We used data from a prospective, community-based mother-and-child cohort (BIRDY, 2012 to 2018) conducted across urban and rural sites in Madagascar, Senegal, and Cambodia. Children were included at birth and followed-up for 3 to 24 months. Data from all outpatient consultations and antibiotics prescriptions were recorded. We defined inappropriate prescriptions as antibiotics prescribed for a health event determined not to require antibiotic therapy (antibiotic duration, dosage, and formulation were not considered). Antibiotic appropriateness was determined a posteriori using a classification algorithm developed according to international clinical guidelines. We used mixed logistic analyses to investigate risk factors for antibiotic prescription during consultations in which children were determined not to require antibiotics. Among the 2,719 children included in this analysis, there were 11,762 outpatient consultations over the follow-up period, of which 3,448 resulted in antibiotic prescription. Overall, 76.5% of consultations resulting in antibiotic prescription were determined not to require antibiotics, ranging from 71.5% in Madagascar to 83.3% in Cambodia. Among the 10,416 consultations (88.6%) determined not to require antibiotic therapy, 25.3% (*n* = 2,639) nonetheless resulted in antibiotic prescription. This proportion was much lower in Madagascar (15.6%) than in Cambodia (57.0%) or Senegal (57.2%) (*p* < 0.001). Among the consultations determined not to require antibiotics, in both Cambodia and Madagascar the diagnoses accounting for the greatest absolute share of inappropriate prescribing were rhinopharyngitis (59.0% of associated consultations in Cambodia, 7.9% in Madagascar) and gastroenteritis without evidence of blood in the stool (61.6% and 24.6%, respectively). In Senegal, uncomplicated bronchiolitis accounted for the greatest number of inappropriate prescriptions (84.4% of associated consultations). Across all inappropriate prescriptions, the most frequently prescribed antibiotic was amoxicillin in Cambodia and Madagascar (42.1% and 29.2%, respectively) and cefixime in Senegal (31.2%). Covariates associated with an increased risk of inappropriate prescription include patient age greater than 3 months (adjusted odds ratios (aOR) with 95% confidence interval (95% CI) ranged across countries from 1.91 [1.63, 2.25] to 5.25 [3.85, 7.15], *p* < 0.001) and living in rural as opposed to urban settings (aOR ranged across countries from 1.83 [1.57, 2.14] to 4.40 [2.34, 8.28], *p* < 0.001). Diagnosis with a higher severity score was also associated with an increased risk of inappropriate prescription (aOR = 2.00 [1.75, 2.30] for moderately severe, 3.10 [2.47, 3.91] for most severe, *p* < 0.001), as was consultation during the rainy season (aOR = 1.32 [1.19, 1.47], *p* < 0.001). The main limitation of our study is the lack of bacteriological documentation, which may have resulted in some diagnosis misclassification and possible overestimation of inappropriate antibiotic prescription.

**Conclusion:**

In this study, we observed extensive inappropriate antibiotic prescribing among pediatric outpatients in Madagascar, Senegal, and Cambodia. Despite great intercountry heterogeneity in prescribing practices, we identified common risk factors for inappropriate prescription. This underscores the importance of implementing local programs to optimize antibiotic prescribing at the community level in LMICs.

## Abstract 2

In a community based cohort study, Bich-Tram Huynh and colleagues investigate the impact of inappropriate antibiotic prescribing on children in Madagascar, Cambodia and Senegal.

## Author summary

**Why was this study done?:**

- Antibiotic resistance is a major public health issue in low- and middle-income countries (LMICs), particularly among children, who face a disproportionately large share of the global burden of bacterial disease.
- Antibiotic overuse, as a result of inappropriate prescribing, is a major driver of antibiotic resistance, but relevant data from LMICs are scarce at the community level, where the majority of antibiotic prescribing occurs.
- Data regarding the prevalence and risk factors of inappropriate prescribing among outpatient children in LMICs are needed to understand its extent and causes and to inform intervention.

**What did the researchers do and find?:**

- Using data from a mother-and-child cohort in urban and rural sites of Madagascar, Senegal, and Cambodia, the appropriateness of outpatient antibiotic prescribing was assessed among all children (*N* = 2,719) having at least 1 outpatient consultation from birth up to 24 months of age.
- We identified that approximately 3 quarters of all antibiotic prescriptions were inappropriate.
- We found that 15.5%, 57.0%, and 57.2% of consultations not requiring antibiotic therapy, nonetheless, resulted in antibiotic prescription in Madagascar, Cambodia, and Senegal, respectively.
- Risk factors for inappropriate prescription include older patient age (being older than 3 months), disease with a higher severity score, consultation during the rainy season, and residence in rural areas.

**What do these findings mean?:**

- Inappropriate antibiotic prescribing is pervasive among outpatient children in LMICs, although there is great heterogeneity across countries in prescribing practices.
- Locally adapted antibiotic stewardship programs are needed to optimize pediatric antibiotic prescribing, while taking into account the specific contexts associated with high-risk populations across diverse LMICs.

## Introduction

Antibiotic resistance is a leading global threat to public health [1]. Over the past 2 decades, there has been an alarming increase in the number of pathogenic bacteria that have developed resistance to common antibacterial agents, particularly in low- and middle-income countries (LMICs), where antibiotic-resistant bacteria appear to be more prevalent than in high-income countries (HICs) [2]. Recent increases in antibiotic resistance in LMICs may be explained by various factors, including changing prescribing patterns among healthcare providers, and increasing patient access both to formal healthcare services and to informal or “over-the-counter” antibiotics. Together, these trends have translated to increases in overall antibiotic consumption in the general community in LMICs over recent years [3,4].

Inappropriate antibiotic prescribing is a primary factor underlying the emergence of antibiotic resistance. An antibiotic prescription may be deemed inappropriate if the chosen dose, molecule, or schedule is nonoptimal, or if the patient’s clinical indication does not necessitate antibiotic therapy. In this study, we focus on the latter. Inappropriate antibiotic prescribing has deleterious impacts on human health across scales. At the individual level, patients are unnecessarily exposed to potential side effects of antibiotic consumption, including allergic reactions and digestive symptoms related to microbiome dysbiosis, and may experience delays to appropriate care. Potential longer-term risks of pediatric antibiotic exposure, such as increased risks of asthma and autoimmune disorders, continue to be described [5]. At the population level, inappropriate antibiotic prescribing creates additional selection pressure favoring the emergence and spread of bacterial strains and serotypes that resist the desired clinical effects of antibiotics [6,7].

In the context of efforts to mitigate the burden of antibiotic resistance, reducing inappropriate antibiotic prescribing is a public health priority. However, the share of antibiotic prescribing that qualifies as inappropriate is poorly described in LMICs. Most studies estimating the magnitude of and risk factors for inappropriate antibiotic prescribing have been based in HICs [8–11]. In particular, there are few studies conducted in LMICs among outpatient children even though a large share of antibiotic prescribing occurs in primary care, and young children in LMICs bear the greatest share of global infectious disease burden and are hence heavily exposed to antibiotics in the first years of life [12,13]. Yet data describing the magnitude and determinants of inappropriate antibiotic prescribing among children in LMICs are lacking. Such data are necessary to quantify potential for prescribing reductions, to describe which populations are potentially overexposed to antibiotics, and ultimately to inform interventions targeting prevention of inappropriate prescribing.

The objectives of this study were to describe and quantify inappropriate antibiotic prescribing and to identify determinants of inappropriate prescribing among children enrolled in a community-based cohort in Madagascar, Cambodia, and Senegal.

## Methods

### Study design

We used data from the BIRDY cohort (Bacterial Infections and antibiotic Resistant Diseases among Young children in LMICs), a prospective, multicentric, community-based mother-and-child cohort. The study took place across urban and rural sites in Madagascar (pilot phase: 2012 to 2014 and complete phase: 2014 to 2018), Senegal (2014 to 2018), and Cambodia (2014 to 2018). The primary objective of this cohort study was to estimate the incidence of antibiotic-resistant bacterial infections in children.

The formal analysis plan for the BIRDY cohort was established in 2012 [14]. There were no changes in the protocol between the pilot phase and complete phase that may bias results of the present study. No additional formal prospective analysis plan specific to this study on inappropriate antibiotic prescribing was made. All methodological details, including development of the classification algorithm for antibiotic appropriateness, definition of outcomes and covariates and statistical methodology, were discussed prior to data analysis in 2021. There were no modifications to this initial planned analysis or main hypotheses subsequent to data exploration. Changes considered subsequent to peer review are described below where appropriate. This study is reported as per the Strengthening the Reporting of Observational Studies in Epidemiology (STROBE) guideline (S1 Checklist).

This BIRDY cohort has been described in detail elsewhere [14]. Briefly, in Cambodia, the study was conducted in 2 urban districts of Phnom Penh (population: 87,035) and 2 rural districts of Kampong Speu (population: 79,000). In Madagascar, the study was conducted in 3 urban districts of Antananarivo (population: 14,997) and in the semirural city of Moramanga (population: 17,159). In Senegal, the study was conducted in an urban district of Guédiawaye (population: 20,529) and in the rural community of Sokone (population: 14,500).

To recruit study participants, a first phase of exhaustive identification of all pregnant women was carried out in the study areas. Women were enrolled, after having given written informed consent, during their third trimester of pregnancy and followed until delivery to include their newborns at birth.

Inclusion criteria were the following for both the mother and her newborn: routine residence in the study area with no plan to move away during the follow-up period, and no opposition to the research being conducted, nor to the collection of epidemiological data and biological samples. Data regarding sociodemographic, medical, and obstetric characteristics of mothers were collected, as well as data regarding the delivery and health characteristics of newborns.

After delivery, children were followed up for between 3 and 24 months, depending on the site and country. Duration of follow-up was 6 months during the pilot phase and 18 months during the complete phase in Madagascar and 24 months in Cambodia. In Senegal, children were followed up for 3 months in rural areas and 12 months in urban areas. Children were followed up by both active and passive surveillance according to the same schedule across all study sites. Active surveillance consisted of scheduled home visits within 3 days of childbirth, followed by weekly visits until the first month of life, fortnightly visits from the first to the third months of life, and finally, monthly visits until the end of the study period. During these visits, children’s health status was checked, especially for signs of infection. In case of a health event, the child was referred to an outpatient consultation or directly to hospital if the child’s condition was serious. Passive surveillance consisted of asking mothers to contact the investigation team whenever the child showed signs of infection in order to schedule an outpatient consultation. Thus, children were able to benefit from outpatient consultations whenever they were sick, whether identified by their guardians or by study investigators. Clinical decisions regarding antibiotic prescribing were not monitored or imposed, but were rather left to the attending physicians to decide according to local protocols.

Importantly, epidemiological data collected during these consultations included children’s diagnoses, symptoms, and treatments received, including details of any antibiotics prescribed. Costs of consultation and antimicrobial treatment were covered by the study.

### Study population and outcomes

For the present study, the population considered was all children from the BIRDY cohort who had at least 1 outpatient consultation during their follow-up. Only systemic antibiotics were taken into account (oral, intramuscular, and intravenous). We did not consider local antibiotics (dermal and ophthalmic) because their impact on antibiotic resistance is probably different from systemic antibiotics.

For each consultation, diagnoses were categorized by the study team using data collected during children consultation. First, we ensured that all diagnoses made by the physician not of infectious origin were concordant with data collected on the signs and symptoms presented by the child. Then, all infectious diagnoses were determined using a syndromic algorithm based on the World Health Organization (WHO) Integrated Management of Childhood Illness (IMCI) guidelines. This algorithm was developed previously and is described elsewhere [15]. All health events, whether infectious or not, that were classified as “uncertain” or not classified by the algorithm were reviewed by a pediatrician (EK) and a medical epidemiologist (BTH).

We defined inappropriate antibiotic prescriptions as those being prescribed during an outpatient consultation for a child’s health event that does not require antibiotic therapy, regardless of the duration of prescription, type of antibiotic, and dosage.

We constructed a standardized classification algorithm in 2 steps to determine which health events require antibiotics or not (S1 Table). The first step was to define diagnoses that do not require antibiotic therapy. For some diagnoses, the indication for antibiotic therapy depends on other discriminating criteria, such as age and/or signs of severity and/or associated symptoms. Thus, the second step was to define criteria to classify the remaining health events that could not be classified solely based upon diagnosis in the first step. These criteria were defined based on the IMCI guidelines and the French pediatric reference manual “Pédiatrie pour le Praticien–médecine de l’enfant et de l’adolescent” [16,17].

Some children received multiple diagnoses and/or multiple antibiotic prescriptions during a single consultation; hence, the number of consultations not requiring antibiotics and the number of antibiotics prescribed among consultations not requiring antibiotics are considered as distinct outcomes. For children receiving multiple diagnoses during their consultation, antibiotic therapy was defined as inappropriate provided that none of the diagnoses required antibiotic therapy. For children receiving prescriptions for multiple antibiotic classes or molecules during a single consultation, all prescriptions were defined as inappropriate provided that none of the corresponding clinical diagnoses required antibiotic therapy.

### Statistical analyses

First, we calculated basic descriptive statistics of characteristics of the study population in each country, including the total number of consultations, diagnoses, antibiotic prescriptions, and inappropriate antibiotic prescriptions. Comparisons between countries were made using the chi2 test or exact Fisher test (depending on the variable’s distribution) for qualitative variables and the Student test or nonparametric Mann–Whitney test (depending on the variable’s distribution) for quantitative variables. All tests were bilateral, and the significance threshold was set at 0.05.

Second, we evaluated risk factors for inappropriate antibiotic prescribing. Specifically, we investigated whether antibiotic prescription during outpatient consultations among children not requiring antibiotic therapy was associated with exposures of primary interest, defined as characteristics of the health event (history of the health event, severity score, season, and child’s age). We considered site, child sex, child nutritional status, and sociodemographic, maternal, and pregnancy characteristics as potential confounders. We included all consultations among children whose health event was determined not to require antibiotic therapy by the classification algorithm described above.

Nutritional status was defined using the z-score, adjusted according to the child’s age and weight using WHO reference tables, where children with a z-score < -2 were considered underweight [18]. When information on the child’s weight at consultation was missing, weight z-score at the closest consultation or visit was used. The severity of each health event was characterized based on clinical signs observed by the physician and according to the WHO IMCI standard [16]. A clinical severity score variable was defined as an ordinal variable with 3 values, ranging from 0 (least severe) to 2 (most severe). House density was defined as the number of people living in the household divided by the number of sleeping rooms in the household. Overcrowding was defined as a house density ≥4, corresponding to the third quartile of the house density variable. We considered the season in which the consultation occurred, defined as rainy versus not. The rainy season was defined as April to September in Cambodia, November to April in Madagascar, and July to November in Senegal. We considered a delivery to be complicated if the child was born by cesarean section, if the child required resuscitation following delivery, or if the delivery was deemed difficult by the practitioner. We considered that the impact of those events would be low after 3 months of age and set this variable to “0” for children older than 3 months.

We used a mixed logistic regression model to account for repeated measures (i.e., multiple consultations for the same child) with a random intercept for each child. Effects of exposures of primary interest on inappropriate antibiotic prescription were assumed to be the same for all children and, accordingly, were modeled as fixed effects. Upon recommendation from a statistical reviewer, we conducted a full model including data from all 3 countries. First, we conducted the full model without any interaction terms (S2 Table). Then, we tested possible interactions between each covariate and the country effect by considering each possible country:covariate interaction term one at a time (S3 Table). As 16 tests were performed, we applied Bonferroni’s correction to counteract inflated type I error. The Bonferroni-corrected alpha-value considered for statistical significance of a variable was calculated as 0.05/16 = 0.003. In the final model, only the interaction terms identified as significant in the previous step were included.

A secondary analysis was also carried out, in which separate mixed logistic regression models were developed for each country to identify risk factors for inappropriate prescribing that are directly reflective of the local contexts (S4 and S5 Tables). For this analysis, both univariable and multivariable analyses were performed. For the multivariable analysis, all fixed-effect variables examined in univariable analysis were included and a backward stepwise Akaike information criterion (AIC) model selection procedure was conducted to select the variables resulting in the model with the lowest AIC. This approach was selected as it has interesting properties, namely that it is asymptotically efficient even if the model is misspecified [19]. All analyses were performed using R software version 4.1.2 (2021-11-01).

### Ethics

The study was approved by the respective ethics committees of Madagascar (068-MSANP/CE), Senegal (SEN 14–20), Cambodia (108 NEHCR), and the Institutional Review Board of Institut Pasteur (IRB/2016/08/03), France. Written informed consent was given by parents of all participants.

## Results

### Study population and consultation characteristics

**Fig 1:**
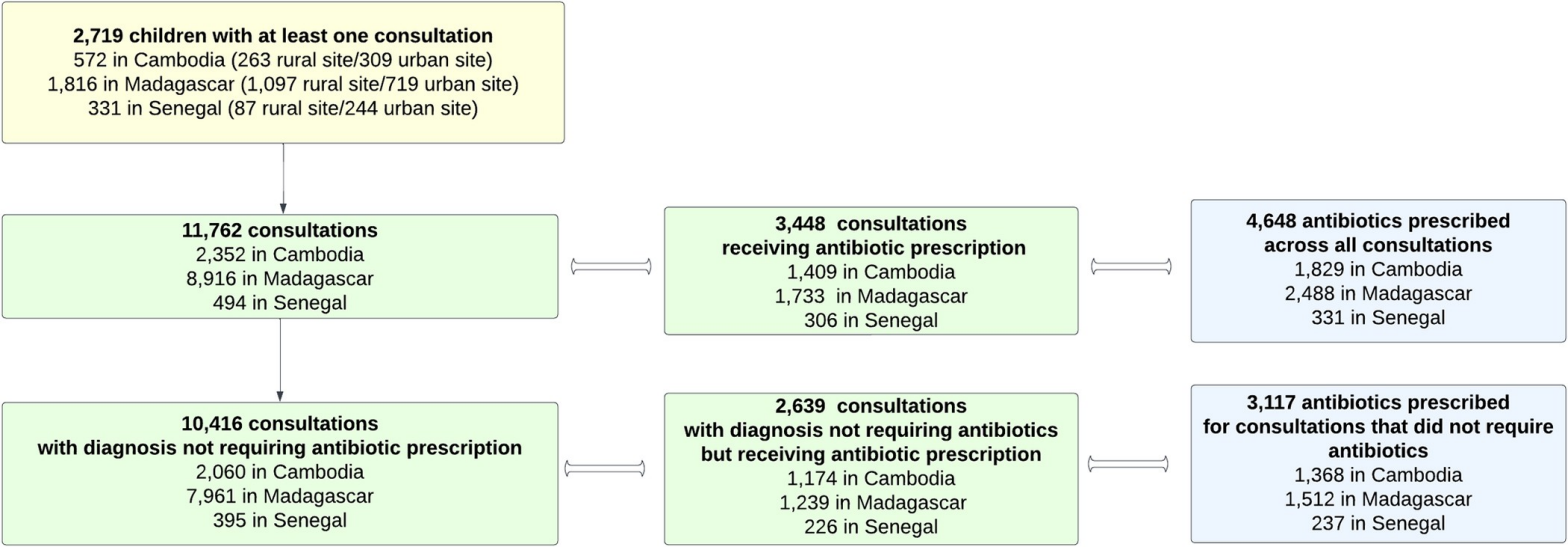
Flow chart of children included in this study (yellow box), their outpatient consultations (green boxes), and their antibiotic prescriptions (blue boxes). The number of antibiotic prescriptions exceeds the number of consultations receiving prescriptions because some consultations resulted in multiple prescriptions.

Among the 3,710 children enrolled at birth between 2012 and 2018, 2,719 (73%) children had at least 1 outpatient medical consultation during their follow-up and were included in this study, including 572 children in Cambodia, 1,816 children in Madagascar, and 331 in Senegal (Fig 1). Overall, 81.6% of included children had a complete follow-up in Cambodia, 94.0% (pilot phase) and 42.9% (complete phase) in Madagascar, and 59.8% (urban site) and 75.9% (rural site) in Senegal. The mean duration of follow-up was 670 days in Cambodia, 160 (pilot phase) and 372 (complete phase) days in Madagascar, and 258 (urban site) and 73 (rural site) days in Senegal.

The proportion of mothers who completed secondary school or attended university was 24.3% in Madagascar, compared to 13.6% in Cambodia and 9.1% in Senegal (*p* < 0.001, Table 1). Most households had electricity in Cambodia (99.5%) and Senegal (98.2%), but just 75.6% of households in Madagascar (*p* < 0.001). Most mothers gave birth in a healthcare facility in Cambodia (98.6%) and Senegal (96.1%), while 37.7% of mothers delivered at home in Madagascar (*p* < 0.001).

**Table 1:** Description of the characteristics of the 2,719 children from the BIRDY cohort who had at least 1 outpatient medical consultation and were hence included in this study.

				Country			
Characteristics				Cambodia *n* (%)	Madagascar *n* (%)	Senegal *n* (%)	*p*-values^μ^
*N* = 2,719				572	1,816	331	
**Site**	Rural			263 (46.0)	1,097 (60.4)	87 (26.3)	<0.001
**Sex**	Male			280 (49.1)	935 (51.5)	166 (50.9)	0.476
*Missing data*				2 (0.3)	0 (0.0)	5 (1.5)	
**Mother’s level of education**							<0.001
None or primary school				314 (54.9)	436 (24.0)	249 (75.2)	
Incomplete secondary				180 (31.5)	939 (51.7)	52 (15.7)	
Secondary or university				78 (13.6)	441 (24.3)	30 (9.1)	
**Mother’s profession**							<0.001
Executive or office job				23 (4.0)	102 (5.6)	6 (1.8)	
Manual job				332 (58.0)	453 (25.0)	81 (24.5)	
Student or unemployed				217 (37.9)	1,260 (69.4)	244 (73.7)	
*Missing data*				0 (0.0)	1 (0.1)	0 (0.0)	
**Mother’s age** (mean (SD)) [range]				27.39 (5.54) [15–46]	26.02 (6.5) [15–48]	28.49 (6.7) [15–46]	<0.001
**Parity**	First child			241 (42.1)	673 (37.1)	77 (23.3)	<0.001
*Missing data*				0 (0.0)	1 (0.1)	0 (0.0)	
**History of deceased child**			Yes	19 (3.3)	130 (7.2)	17 (5.1)	0.001
*Missing data*				0 (0.0)	1 (0.1)	0 (0.0)	
**Latrine**	Exterior			272 (47.6)	1,679 (92.5)	193 (58.7)	<0.001
*Missing data*				0 (0.0)	1 (0.1)	2 (0.6)	
**Electricity**	Yes			569 (99.5)	1,373 (75.6)	324 (98.2)	<0.001
*Missing data*				0 (0.0)	1 (0.1)	1 (0.3)	
**Density of the house**		Overcrowded		274 (47.9)	625 (34.4)	110 (33.2)	<0.001
*Missing data*				1 (0.2)	1 (0.1)	5 (1.5)	
**Place of delivery**	Health facility			563 (98.6)	1,132 (62.3)	317 (96.1)	<0.001
*Missing data*				1 (0.2)	0 (0.0)	1 (0.3)	
**Cesarean section**	Yes			71 (12.5)	180 (9.9)	11 (3.4)	<0.001
*Missing data*				2 (0.3)	0 (0.0)	8 (2.4)	
**Resuscitation at birth**							0.268
No				518 (90.6)	1,628 (89.6)	292 (88.2)	
Yes				49 (8.6)	183 (10.1)	21 (6.3)	
*Missing data*				5 (0.8)	5 (0.3)	18 (5.5)	
**Dystocic delivery**							<0.001
No				524 (91.6)	1,625 (89.5)	278 (84.0)	
Yes				33 (5.8)	178 (9.8)	6 (1.8)	
*Missing data*				15 (2.6)	13 (0.7)	47 (14.2)	
**Low birth weight** *							0.002
No				538 (94.1)	1,642 (90.4)	257 (77.6)	
Yes				26 (4.5)	160 (8.8)	31 (9.4)	
*Missing data*				8 (1.4)	14 (0.8)	43 (13.0)	
**Premature delivery** ^$^							0.123
No				544 (95.1)	1,691 (93.1)	280 (84.6)	
Yes				19 (3.3)	66 (3.6)	5 (1.5)	
*Missing data*				9 (1.6)	59 (3.2)	46 (13.9)	

*Low birth weight is defined as birth weight <2,500 g.

^**$**^Birth before 37 weeks gestation.

^μ^*p*-values were calculated based on non-missing data and using Chi-square tests for categorical variables and F-tests for continuous variables.

Among the 2,719 children having at least 1 consultation, a total of 11,762 outpatient consultations occurred over the follow-up period. Among these, 3,448 (29.3%) consultations led to antibiotic prescriptions. These consultations represented 59.9% (1,409/2,352), 19.4% (1,733/8,916), and 61.9% (306/494) of all consultations in Cambodia, Madagascar, and Senegal, respectively (Fig 1).

In both Cambodia and Madagascar, the most frequent diagnosis was rhinopharyngitis, representing 34.6% (814/2,352) and 29.5% (2,634/8,916) of consultations, respectively. Gastroenteritis was the next most common diagnosis, accounting for 17.6% (416/2,352) of consultations in Cambodia and 12.5% (1,112/8,916) in Madagascar. The third most common diagnosis was bronchiolitis in Cambodia, with 8.9% (210/2,352) of consultations, and lower respiratory infection in Madagascar, with 10.8% (964/8,916) of consultations. In Senegal, the 3 most common diagnoses were bronchiolitis with 15.6% of consultations (77/494), healthy child (consultations without specific diagnoses/symptoms) with 13.3% of consultations (66/494), and rhinopharyngitis with 10.9% of consultations (54/494).

Across all consultations that led to antibiotic prescriptions, a total of 4,648 distinct antibiotics were prescribed, as some prescriptions included multiple antibiotics. The most prescribed antibiotic in Cambodia and Madagascar was amoxicillin, accounting for 35.7% (653/1,829) and 24.0% (597/2,488) of all antibiotics prescribed, respectively. Cefixime was the most prescribed antibiotic in Senegal, representing 34.4% (114/331) of all antibiotics prescribed (S6 Table).

### Inappropriate antibiotic prescriptions

Of the 11,762 outpatient consultations, 10,416 were associated with diagnoses (88.6%) considered not to require antibiotic therapy. Of these, 25.3% (2,639/10,416, 95% CI [24.5, 26.2]) led to an antibiotic prescription. This proportion differed greatly among the 3 countries: 57.0% (1,174/2,060, 95% CI [54.8, 59.1]) in Cambodia, 15.6% (1,239/7,961, 95% CI [14.7, 16.4]) in Madagascar, and 57.2% (226/395, 95% CI [52.2, 62.2]) in Senegal (*p* < 0.001). Altogether, 76.5% (2,639/3,448, 95% CI [75.1, 77.9]) of all outpatient consultations resulting in antibiotic prescriptions did not require them, including 83.3% (1,174/1,409, 95% CI [81.3, 85.2]) in Cambodia, 71.5% (1,239/1,733, 95% CI [69.3, 73.6]) in Madagascar, and 73.9% (226/306, 95% CI [68.6, 78.7]) in Senegal (*p* < 0.001). If we consider distinct antibiotic prescriptions, altogether 67.1% (3,117/4,648, 95% CI [65.7, 68.4]) of all prescriptions were inappropriate, including 74.8% (1,368/1,829, 95% CI [72.7, 76.7]) in Cambodia, 60.8% (1,512/2,488, 95% CI [58.8, 62.7]) in Madagascar, and 71.6% (237/331, 95% CI [66.4, 76.4]) in Senegal (*p* < 0.001) (Fig 1).

**Fig 2:**
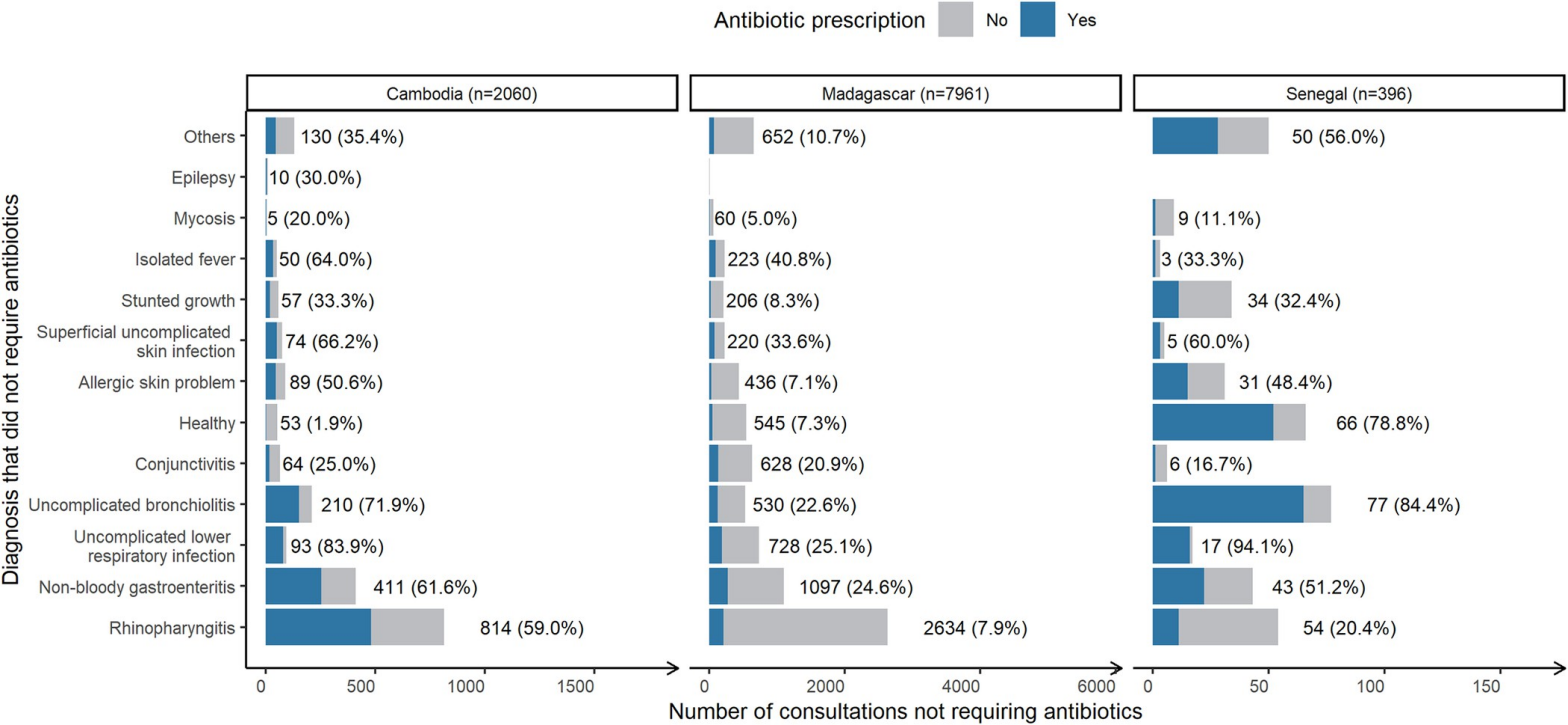
Number of consultations “not requiring antibiotics” stratified by country, associated diagnosis and the share resulting in antibiotic prescription (blue). Across all countries, *N* = 10,416. *Number of consultations with this diagnosis (percentage resulting in antibiotic prescription).

Among the consultations determined not to require antibiotics, the diagnoses accounting for the greatest absolute share of inappropriate prescribing in Cambodia were rhinopharyngitis, with 480 of 814 (59.0%) associated consultations resulting in prescriptions and non-bloody gastroenteritis with 253 of 411 consultations (61.6%) (Fig 2). In Madagascar, these diagnoses also accounted for the greatest share of inappropriate prescribing, with antibiotic prescriptions resulting from 209 of 2,634 (7.9%) rhinopharyngitis consultations and 270 of 1,097 (24.6%) non-bloody gastroenteritis consultations. In Senegal, uncomplicated bronchiolitis accounted for the greatest absolute number of inappropriate prescriptions at 65 of 77 (84.4%) consultations. The proportion of consultations in which children were prescribed antibiotics despite being diagnosed as in “good health” was 1.9% in Cambodia (1/53), 7.3% in Madagascar (40/545), and 78.8% in Senegal (52/66).

**Fig 3:**
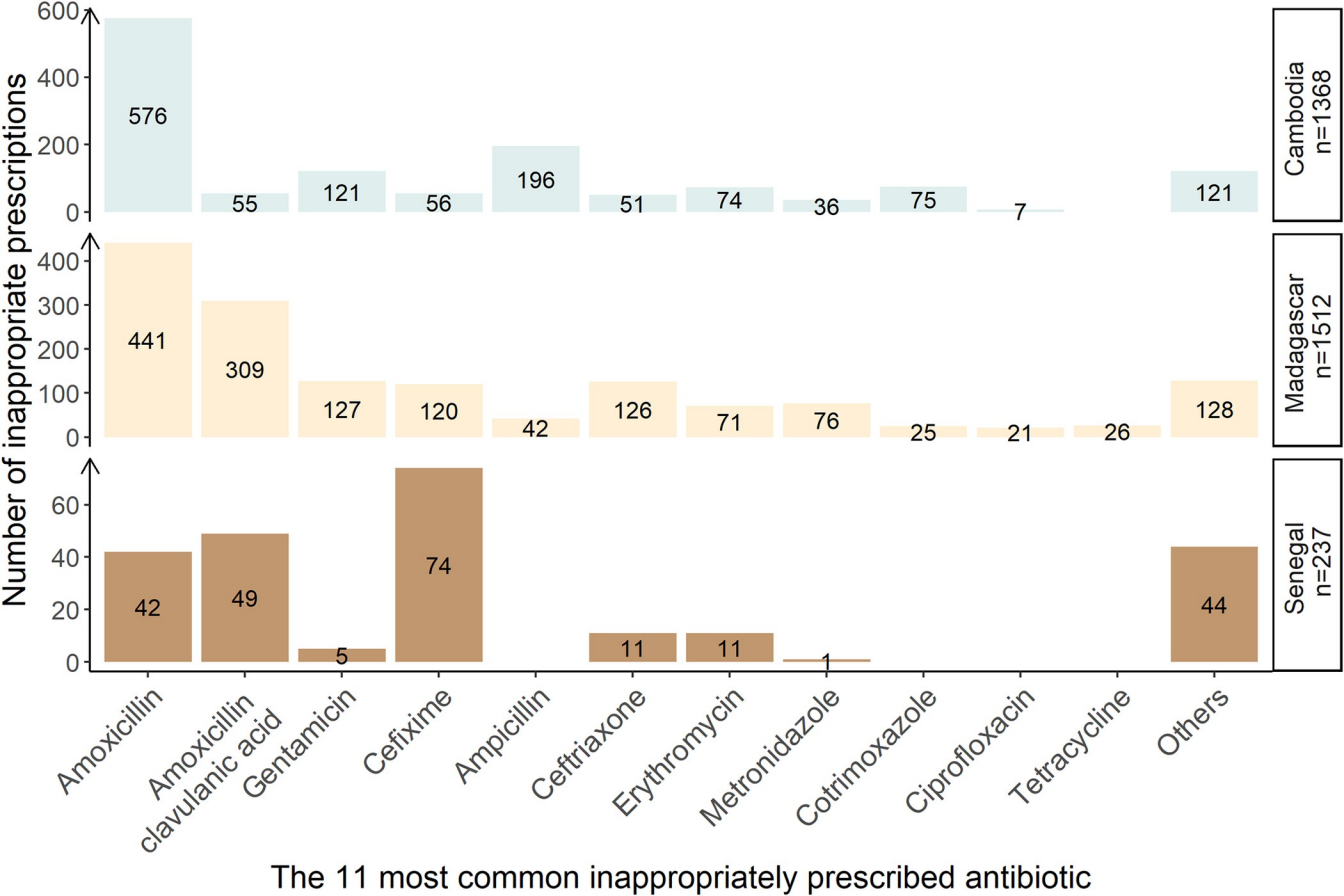
Number of antibiotics prescribed for diagnoses that did not require antibiotics, stratified by country and type of antibiotic. *N* = 3,117 distinct antibiotics prescribed.

The most frequently prescribed inappropriate antibiotic was amoxicillin in Cambodia and Madagascar, accounting for 42.1% (576/1,368) and 29.2% (441/1,512) of inappropriate prescriptions, respectively. In Senegal, cefixime was the antibiotic most frequently prescribed inappropriately, representing 31.2% of inappropriate prescriptions (74/237) (Fig 3).

### Determinants of inappropriate antibiotic prescribing

Table 2 describes determinants of antibiotic prescribing among consultations with a diagnosis determined not to require antibiotic therapy. Covariates with significant country-level effects (i.e., significant country:covariate interactions) were age and site (S3 Table).

**Table 2:** Analysis of the determinants of antibiotic prescription among consultations of children of the 3 countries (Cambodia, Madagascar, and Senegal) with a diagnosis not requiring antibiotic therapy.

		Madagascar, Cambodia, and Senegal *n* = 10,336 consultations among 2,509 children*	
Variables		Adjusted OR (95% CI)	P^1^
**Age** ^2^	<3 mo	ref	**<0.001**
3 mo–1 yr		4.51 (3.34–6.08)	
>1 yr		5.25 (3.85–7.15)	
**Weight z-score**	Normal	ref	0.670
Underweight		1.03 (0.87–1.22)	
**History of hospitalization in the last 90 days** = Yes		0.94 (0.75–1.18)	0.628
**History of antibiotic prescription in the last 15 days** = Yes		**1.50** (1.16–1.94)	**0.002**
**Severity score**	0	Ref	**<0.001**
1		**2.00** (1.75–2.30)	
2		**3.10** (2.47–3.91)	
**Season =** Rainy		**1.32** (1.19–1.47)	**<0.001**
**Complicated delivery** = Yes		1.09 (0.85–1.39)	0.487
**Country**	Cambodia	ref	**<0.001**
Madagascar		**0.28** (0.20–0.38)	
Senegal		**2.60** (1.67–4.04)	
**Site** = Rural		**4.07** (3.12–5.31)	**<0.001**
**Sex** = Male		1.08 (0.97–1.21)	0.181
**Mother’s level of education**			
None or primary school		ref	0.576
Incomplete secondary		0.89 (0.78–1.02)	
Secondary or university		0.89 (0.74–1.06)	
**Mother’s age**	<26 yrs	1.10 (0.96–1.25)	0.168
**Mother’s profession**	Manual	ref	0.570
Executive or office job		0.90 (0.67–1.20)	
Student or unemployed		0.94 (0.82–1.07)	
**Parity**	First child	0.94 (0.81–1.08)	0.393
**History of deceased child**	Yes	0.98 (0.78–1.24)	0.859
**House density**	Normal	ref	0.012
Overcrowded (4 or more)		**1.17** (1.03–1.32)	
**Place of delivery**	Health facility	ref	0.103
At home		1.13 (0.98–1.31)	
**Site x Country (Reference: for site: urban and for country: Cambodia**			**<0.001**
Rural: Madagascar		**0.45** (0.33–0.61)	
Rural: Senegal		**1.08** (0.54–2.14)	
**Age x Country Reference: for age: <3 mo and for country: Cambodia**			**<0.001**
3 mo–1 yr: Madagascar		**0.42** (0.31–0.58)	
3 mo–1 yr: Senegal		**0.74** (0.40–1.36)	
>1 yr: Madagascar		**0.69** (0.48–0.99)	
>1 yr: Senegal		NA	
**Country adjusted ORs taking into account interaction parameters** ^3^			
**Site** = Rural			
Cambodia		**4.07** (3.12–5.31)	
Madagascar		**1.83** (1.57–2.14)	
Senegal		**4.40** (2.34–8.28)	
**Age** ^2^			
3 mo–1 yr			
Cambodia		**4.51** (3.34–6.08)	
Madagascar		**1.91** (1.63–2.25)	
Senegal		**3.34** (1.95–5.75)	
>1 yr			
Cambodia		**5.25** (3.85–7.15)	
Madagascar		**3.65** (2.96–4.48)	
Senegal		NA	

^1^*P*-values are calculated from individual Wald tests. The threshold for *p*-values significance was set at 0.003 after Bonferroni correction.

^2^In Senegal, ORs associated with age at consultation between 3 months and 1 year should be interpreted only for infants of the urban site (no infants >3 months included in the rural site) and ORs associated with age at consultation >1 year should not be interpreted (no infants >1 year included).

^3^Adjusted ORs were calculated by considering both fixed and interaction effects for Madagascar and Senegal. Confidence intervals were obtained by considering both the variances of the estimates and the covariance between them.

*Analysis performed on consultations with complete dataset (for details, see S5 Table).

Ref, reference; OR, odds ratio; 95% CI, 95% confidence interval; NA, Not Applicable.

We found that older age at consultation was associated with higher risk of receiving inappropriate antibiotic prescription in all 3 countries. Children aged >3 months had higher risk of inappropriate prescription compared to children aged <3 months, with adjusted odds ratios (aORs) for children aged 3 months to 1 year estimated at 1.91 (95% CI [1.63, 2.25]; *p* < 0.001), 3.34 (95% CI [1.95, 5.75]; *p* < 0.001), and 4.51 (95% CI [3.34, 6.08]; *p* < 0.001) in Madagascar, Senegal, and Cambodia, respectively. For children followed up beyond 1 year of age, aORs were 3.65 (95% CI [2.96, 4.28]; *p* < 0.001) and 5.25 (95% CI [3.85, 7.15]; *p* < 0.001) in Madagascar and Cambodia, respectively.

Consultations during the rainy season were associated with higher risk of inappropriate antibiotic prescription (aOR = 1.26, 95% CI [1.32, 1.47]; *p* < 0.001). Children living in rural areas had a higher risk of inappropriate prescription compared to those living in urban areas, with aORs of 1.83 (95% CI [1.57, 2.14]; *p* < 0.001) in Madagascar, 4.40 (95% CI [2.34, 8.28]; *p* < 0.001) in Senegal, and 4.07 (95% CI [3.12, 5.31]; *p* < 0.001) in Cambodia.

Having already received an antibiotic prescription in the 15 days prior to the consultation also increased the risk of inappropriate prescription (aOR = 1.50; 95% CI [1.16, 1.94]; *p* = 0.002). Finally, children with higher clinical severity score had a higher risk of inappropriate antibiotic prescription compared to children with a lower score, with aORs ranging from 2.00 (95% CI [1.75, 2.30]) to 3.10 (95% CI [2.47, 3.91]) for children with severity scores of = 1 or = 2, respectively, relative to severity score = 0.

In the secondary analysis considering a distinct model per country, results were highly similar. The 4 variables that were identified as relevant across each of the 3 countries (age at consultation, season, site, and severity score) were also identified as statistically significant in the primary analysis considering a single model (S4 Table).

## Discussion

In this multicentric community cohort study conducted among children under 2 years old in Madagascar, Cambodia, and Senegal, we observed that approximately 1 quarter of outpatient consultations not requiring antibiotic therapy nonetheless resulted in an antibiotic prescription. Overall, approximately 3 quarters of consultations resulting in antibiotic prescription did not require them, and approximately two thirds of all antibiotics prescribed to these young children were not necessary. These inappropriate antibiotics may contribute to the emergence of antibiotic resistance despite providing little to no benefit—or even harm—to the patients concerned.

Our results suggest that inappropriate antibiotic prescribing is frequent but highly heterogeneous across countries. In particular, approximately 57% of consultations not requiring antibiotics resulted in antibiotic prescriptions in Cambodia and Senegal, but only 16% in Madagascar. Although relatively few previous studies have estimated the share of inappropriate antibiotic prescribing in LMICs, substantial heterogeneity across countries is nonetheless consistent with the literature. In a secondary data analysis study, Sulis and colleagues estimated the proportion of patient–provider interactions resulting in inappropriate antibiotic prescriptions in 3 LMICs (India, China, and Kenya). They found that approximately 50% of interactions in India and Kenya resulted in inappropriate prescriptions, compared to approximately 29% in China [20]. However, this study considered both children and adults and only included certain preselected diagnoses preventing direct comparison with our study, which included all diagnoses among young children only. Our findings thus describe inappropriate antibiotic prescribing across all outpatient health events, resulting in a more complete and representative assessment of local prescribing practices. For this reason, diagnosis-specific estimates of inappropriate antibiotic prescribing may be more easily compared with the literature.

For children with gastroenteritis not requiring antibiotics, we found that the share of consultations resulting in antibiotic prescription was 24.6%, 51.2%, and 61.6% in Madagascar, Senegal, and Cambodia, respectively. In Senegal, our estimates can be compared to those of a study conducted in children under 5 with gastroenteritis in a primary healthcare in Dakar (the capital city of Senegal). In this study, in which all episodes were microbiologically documented, the authors found that 36% of children received antibiotics when no pathogens were detected in the stool [21]. In a study conducted in Madagascar’s capital city, Antananarivo, approximately 47.2% of children with diarrhea received an antibiotic prescription. However, the authors did not distinguish between diarrheal episodes that required antibiotics and those that did not. In this study, physicians were found to justify antibiotic prescribing mainly by the presence of fever [22]. According to WHO, the prescription of antibiotics during an episode of diarrhea is indicated in the presence of bloody diarrhea or documented shigellosis. In such cases, diarrhea is frequently accompanied by fever, but fever is likely to accompany diarrhea regardless of bacterial or viral etiology [16]. One possible explanation for our results is that prescribers had similar practices, as fever was present in 47.8%, 41.1%, and 31.8% of gastroenteritis consultations resulting in an inappropriate prescription in Madagascar, Cambodia, and Senegal, respectively. In a separate study of children <2 years old consulting primary care in LMICs, Rogawski and colleagues estimated that 44.2% of non-bloody diarrhea consultations resulted in antibiotic prescribing, with estimates ranging from 9% to 57% across the 8 countries considered [23]. Finally, Sulis and colleagues estimated that the share of pediatric diarrhea consultations resulting in inappropriate antibiotic prescription ranged from 27.4% in China to 59.4% in India. Overall, these results support the extensive intercountry heterogeneity observed in our study.

For respiratory illness (upper and lower combined) not requiring antibiotics, Rogawski and colleagues further estimated that between 20.2% and 62.2% of consultations, depending on the country, resulted in inappropriate prescription [23]. By comparison, we found that approximately 1 quarter of uncomplicated lower respiratory infection (mainly bronchitis) consultations resulted in inappropriate prescriptions in Madagascar, but over 80% in Cambodia and Senegal. For rhinopharyngitis consultations, we found that 7.9%, 20.4%, and 59.0% resulted in inappropriate antibiotic prescription in Madagascar, Cambodia, and Senegal, respectively. These estimates are consistent with the extensive heterogeneity between countries observed by Rogawski and colleagues, with very high inappropriate antibiotic prescribing for respiratory illness in certain countries [23]. This heterogeneity may be explained by various factors, including local care-seeking behaviors and prescribing practices, as well as variation in the underlying epidemiological burden of disease.

In Senegal, one striking finding is that consultations reported as “healthy” represented one of the most common diagnoses. Reviewing these diagnoses in detail, we found that all consultations reported as “healthy” took place at birth and occurred mainly in the rural site (87%). In addition, there was only 1 “healthy” consultation per child. This seems to be a pattern that is specific to Senegal, reflecting local consultation behavior or prescribing practices, and this result should therefore be interpreted in that specific context. Further studies should be conducted to better understand motivations of both mothers to consult for their newborn at delivery and physicians to prescribe antibiotics despite no clear indication.

Our study also provides information on all-cause antibiotic exposure (regardless of diagnosis or symptoms), which has been more frequently described in the literature. A recent meta-analysis evaluated all-cause antibiotic prescribing across all age groups in primary care in LMICs, estimating that 44% to 60% of consultations resulted in antibiotic prescriptions across 48 studies [12]. However, the authors highlight extensive heterogeneity between studies, which is mainly explained by differences in study design and makes comparison difficult. In our study, all-cause antibiotic prescribing ranged from approximately 20% of consultations in Madagascar to 60% of consultations in Cambodia and Senegal. Stark differences observed between Madagascar and the 2 other countries are not readily explained by different study designs (the protocol was identical for all 3 countries) nor by substantial differences in infectious disease burden (the types and distributions of diagnoses seem to be relatively similar).

By contrast, choice of antibiotic was more similar between Cambodia and Madagascar than Senegal. In particular, the most prescribed antibiotic was amoxicillin in Madagascar and Cambodia (24.0% and 35.7%, respectively), but cefixime (34.4%) in Senegal. Padget and colleagues conducted face-to-face interviews with caregivers of 1,401 children living in the same urban and rural sites in Madagascar and the same urban site in Senegal as in our study and examined antibiotic consumption in the community [24]. Although amoxicillin was the most used antibiotic in all study areas, the authors found that cephalosporins represented 15.9% of antibiotics consumed in the urban site in Senegal compared to 3.5% and 4.5% in the urban and rural sites in Madagascar, respectively. Our results are therefore consistent with these previous findings that cephalosporins (including cefixime) are more widely used in Senegal, possibly reflecting differences in prescribing habits of healthcare providers across these settings.

A study on the sale of antibiotics across low-, middle-, and high-income countries suggests that extensive intercountry variation in both the quantity and classes of antibiotics sold cannot be explained solely by differences in disease burden [25]. Rather, differences may in large part reflect heterogeneity in local healthcare practices, culture, and socioeconomic contexts. However, this analysis was based on drug sales data, thus providing limited information on prescribing practices.

Another recent spatial modeling study provided estimates of global antibiotic consumption based on data from Demographic Health Surveys and UNICEF’s Multiple Indicator Cluster Surveys [26]. The authors have also shown wide variation in antibiotic consumption, both between and within countries. Lack of access to healthcare, particularly in poor and rural communities, as well as particular cultural and socioeconomic contexts might explain these disparities. However, as acknowledged by the authors, although both data sources used in this modeling study provide highly useful data in LMICs, they are subject to biases that may limit the interpretability of the results of this study. Using reliable, prospective, standardized cohort data that facilitates comparisons across sites and countries, our findings thus suggest disparities in antibiotic exposure among children across 3 LMICs.

In this study, we also identified leading determinants of inappropriate antibiotic prescribing. We found that children older than 3 months had a higher risk of receiving an inappropriate prescription than younger infants. This may be explained by a higher risk of severe bacterial infection during the postnatal period and the fact that several diagnoses that are more common in these youngest children require antibiotics [27–30]. This effect of age is consistent with findings from a study of children presenting with suspected pneumonia to a primary healthcare facility in Zambia, which found a higher proportion of incorrect antibiotic prescriptions for children aged 12 to 59 months than children under 12 months [31]. However, the authors did not identify any factors explaining this result.

Clinical severity score was also associated with a higher risk of inappropriate prescription. During consultations in which patients express more frequent or severe symptoms, parents or guardians may be more likely to demand antibiotics. During such consultations, practitioners may also choose to prescribe antibiotics due to concern about the risk of bacterial superinfection. These factors may be particularly relevant for certain diagnoses with extensive symptom expression and known risk of superinfection, such as bronchiolitis, which received a large share of inappropriate prescriptions in this study.

We found that consultations occurring in the rainy season were more likely to result in inappropriate prescriptions than those in the dry season. During rainy periods, respiratory and fecal-oral infections generally circulate more widely, which may have an impact on practitioners’ prescribing practices. If the overall number of consultations for infectious pathologies increases, the practitioner’s choice to prescribe an antibiotic during subsequent consultations may be influenced. Although increases in the total volume of antibiotic prescribing are well observed in winter months in HICs and have been documented in the rainy season in LMICs, seasonality in inappropriate antibiotic prescribing is less well documented, particularly in LMICs [32–34].

We found that children living in rural areas were at increased risk of receiving inappropriate antibiotic prescriptions. This may be explained by a limited presence of qualified health professionals in rural areas, especially physicians. Indeed, several studies have shown that rural areas suffer from a form of medical staff desertification, where public sector physicians initially work in rural areas after graduating from medical school but later move to urban areas [35,36]. Thus, in some instances, the medical staff that remains in these areas are individuals with backgrounds in healthcare but who are not qualified as physicians. Decreased access to healthcare facilities in rural areas may also explain this increased risk, particularly in the context of a practitioner’s potential concern regarding bacterial superinfection, as patients in rural settings may be less able to re-consult in the days following an initial consultation. This is consistent with previous findings from Madagascar by Padget and colleagues, who reported that antibiotic prescriptions increase overall as the density of healthcare facilities decrease [24].

Our results suggest a need for interventions to prevent the inappropriate prescribing of antibiotics to children in LMICs. One possible intervention is to train prescribers with validated pediatric clinical management guidelines. For example, training practitioners with IMCI guidelines has been shown previously to decrease inappropriate antibiotic prescribing in Tanzania and Uganda [37]. Also, lack of access to microbiological documentation in LMICs may explain frequent antibiotic prescription in case of doubt of bacterial origin of the disease. Rapid diagnostic testing may also help prescribers, especially those lacking training or practicing in remote areas, to understand whether a disease is likely to be of viral rather than bacterial etiology. For example, rapid tests are widely used in HICs to identify sore throat due to group A *Streptococcus* and may help to prevent inappropriate antibiotic prescribing by suggesting when viral origin may be more likely [38]. For bronchiolitis, rapid tests to identify respiratory syncytial virus have also demonstrated good specificity, although their use in routine practice remains to be evaluated [39]. C-reactive-protein point-of-care tests may also prove beneficial to improve antibiotic prescription, although optimal cut-off thresholds to distinguish between bacterial and nonbacterial infection across diverse use cases remain to be determined [40,41].

To our knowledge, our study is the first in LMICs to estimate the proportion of outpatient consultations resulting in inappropriate antibiotic prescriptions in young children. It is also the first to estimate the total share of antibiotic prescriptions that are inappropriate in these settings. Our prospective, longitudinal study design with rigorous documentation of patient symptoms and diagnoses facilitated the evaluation of antibiotic necessity for each consultation. This makes our findings a good reflection of local practices regarding both inappropriate and overall antibiotic prescribing, particularly as over-the-counter antibiotics have been shown to represent only a small portion of antibiotic consumption in children [24].

Our study also has several limitations. Bacteriological investigations were performed only in prespecified cases, therefore, not all infectious diagnoses were microbiologically confirmed, and in particular, those presumed to be of viral origin [14]. This may have resulted in misclassification of some diagnoses, including both those that we determined as requiring and not requiring antibiotics. We are unable to estimate the magnitude of these misclassifications, but they may have resulted in overestimation of inappropriate prescribing, given that the proportion of diagnoses not requiring antibiotics is much greater than those not (88.6% versus 11.4%). However, the study protocol provided access to laboratory tests as needed for all children included, at the clinician’s discretion, to guide prescription [14]. We are also unable to assess how representative our results are of antibiotic prescribing in these settings in non-study conditions, in particular, because all monetary costs related to consultations and antibiotic prescriptions were supported by the project. Given this economic context, it is unlikely that children consulted external practitioners during the study period, suggesting that consultation histories should be relatively complete, although this may possibly have resulted in children consulting more frequently than they would normally in non-study conditions. As there were also no financial barriers to prescribing, it is possible that clinicians in our study prescribed antibiotics more frequently than they would in non-study conditions. Further, antibiotic access is known to influence antibiotic use, and reduced barriers to antibiotic access among our study participants may have led to increased probability of prescribed antibiotics being dispensed via formal channels (i.e., pharmacies) and thus captured by our study, and decreased probability of patients accessing informal or over-the-counter antibiotics [42–44].

Depending on the availability of particular healthcare providers at the time of the health event, as well as potential parental preferences, children could consult healthcare providers from either the public or private sectors. This could potentially bias findings as private sector providers may receive economic incentives to prescribe certain types of antibiotics [20]. Finally, our results cannot be generalized to the entire population because our study only concerned children under 2 years of age and included limited sites per country. In particular, the relatively small sample size in Senegal, which was limited due to logistical issues, may limit interpretation of results from this country. Despite these limitations, and in the context of considerable challenges to quantifying and evaluating the appropriateness of community antibiotic prescribing in LMICs, if interpreted with care, we believe that our study has facilitated reliable estimation of trends of inappropriate antibiotic prescribing among young children in these 3 LMICs.

In conclusion, in a community-based cohort of young children spanning 3 LMICs, we observed that the majority of all outpatient antibiotic prescriptions were inappropriate. While the proportion of consultations resulting in inappropriate prescriptions was variable across countries, several common risk factors for inappropriate prescribing were nonetheless identified. These findings support the expansion of interventions to guide antibiotic prescribing that have already proven effective in certain LMIC settings, including training with validated clinical practice guidelines (e.g., IMCI) and the use of rapid diagnostic tests. The decision-making process underlying antibiotic prescription is complex, particularly in the absence of robust microbiological information and given challenging socioeconomic contexts, requiring the implementation of locally adapted multimodal strategies [45–47]. Future research is needed to better understand practitioners’ motivations for prescribing and why they may deviate from established guidelines. Sociological studies and qualitative methods such as practitioner and patient interviews may facilitate a better understanding of antibiotic prescribing and its perceived necessity among high-risk populations in LMICs.

## Supporting information

S1 Checklist Strengthening the Reporting of Observational Studies in Epidemiology (STROBE) checklist. (DOCX)

S1 Table List of diagnoses defined as “not requiring antibiotic therapy,” including diagnoses classified by the two-step algorithm to determine which health events do not require antibiotics in the absence of discriminating criteria. (DOCX)

S2 Table Analysis of the determinants of antibiotic prescription among consultations of children of the 3 countries (Cambodia, Madagascar, and Senegal) with a diagnosis not requiring antibiotic therapy without any covariate: country interaction terms. (DOCX)

S3 Table Results (χ2, degrees of freedom, *p*-values) of including covariate:country interaction terms in the regression model, where each interaction term is considered one at a time. (DOCX)

S4 Table Analysis of the determinants of antibiotic prescription among consultations with a diagnosis not requiring antibiotic therapy, with a separate model considered for each country. (DOCX)

S5 Table Characteristics of consultations determined not to require antibiotic therapy. (DOCX)

S6 Table Distribution of antibiotics prescribed across all consultations, stratified by country. *N* = 4,648 antibiotic prescriptions across all countries.
(DOCX)

## Abbreviations

AIC: Akaike information criterion
aOR: adjusted odds ratio
CI: confidence interval
HIC: high-income country
IMCI: Integrated Management of Childhood Illness
LMIC: low- and middle-income country
WHO: World Health Organization
