# Supplementary material for: Inappropriate antibiotic prescribing and its determinants among outpatient children in 3 low- and middle-income countries: A multicentric community-based cohort study
Source: PLoS Med. 2023 Jun 6;20(6):e1004211. doi: 10.1371/journal.pmed.1004211 (PMC10243627; doi:10.1371/journal.pmed.1004211)
Supplement: S1 Checklist — (DOCX) [file pmed.1004211.s001.docx]

**S1 Checklist**

STROBE Statement—Checklist of items that should be included in reports of ***cohort studies***

|  | **Item No** | **Recommendation** |
| --- | --- | --- |
| **Title and abstract** | 1 | (*a*) Indicate the study’s design with a commonly used term in the title or the abstract  **Title:** “a multicentric community-based cohort study” |
|  |  | (*b*) Provide in the abstract an informative and balanced summary of what was done and what was found  **-Section abstract, sub-section: methods and findings, paragraphs 1-4**  **-Section abstract, sub- section: conclusion** |
| **Introduction** | | |
| Background/rationale | 2 | Explain the scientific background and rationale for the investigation being reported  **Section introduction: paragraphs 1-3** |
| Objectives | 3 | State specific objectives, including any prespecified hypotheses  **Section introduction: paragraph 4** |
| **Methods** | | |
| Study design | 4 | Present key elements of study design early in the paper  **Section methods, subsection: study design, paragraph 1** |
| Setting | 5 | Describe the setting, locations, and relevant dates, including periods of recruitment, exposure, follow-up, and data collection  **Section methods, subsection: study design, paragraphs 1 and 3** |
| Participants | 6 | (*a*) Give the eligibility criteria, and the sources and methods of selection of participants. Describe methods of follow-up  **Section methods, subsection: study design, paragraphs 4-6** |
|  |  | (*b*) For matched studies, give matching criteria and number of exposed and unexposed **NA** |
| Variables | 7 | Clearly define all outcomes, exposures, predictors, potential confounders, and effect modifiers. Give diagnostic criteria, if applicable  **Section methods, subsection: study population and outcomes, S1 table**  **Section methods, subsection: statistical analysis, paragraphs 2-3** |
| Data sources/ measurement | 8* | For each variable of interest, give sources of data and details of methods of assessment (measurement). Describe comparability of assessment methods if there is more than one group  **Section methods, subsection: study design, paragraph 6**  **S1 table**  **Section methods, subsection: study population and outcomes** |
| Bias | 9 | Describe any efforts to address potential sources of bias  **Section methods, subsection: study design, paragraph 7**  **Section methods, subsection: study population and outcomes, paragraph 3**  **Section methods, subsection: statistical analysis, paragraph 2** |
| Study size | 10 | Explain how the study size was arrived at  **Section methods, subsection: Study population and outcomes, paragraph 1**  **Figure 1** |
| Quantitative variables | 11 | Explain how quantitative variables were handled in the analyses. If applicable, describe which groupings were chosen and why  **Section methods, subsection: statistical analysis, paragraph 3** |
| Statistical methods | 12 | (*a*) Describe all statistical methods, including those used to control for confounding  **Section methods, subsection: statistical analysis** |
|  |  | (*b*) Describe any methods used to examine subgroups and interactions  **Section methods, subsection: statistical analysis** |
|  |  | (*c*) Explain how missing data were addressed  **Section methods, subsection: statistical analysis, paragraph 3** |
|  |  | (*d*) If applicable, explain how loss to follow-up was addressed **NA** |
|  |  | (*e*) Describe any sensitivity analyses: **NA** |
| **Results** | | |
| Participants | 13* | (a) Report numbers of individuals at each stage of study—eg numbers potentially eligible, examined for eligibility, confirmed eligible, included in the study, completing follow-up, and analysed  **Section results, subsection: study population and consultations characteristics, paragraph 1**  **Figure 1** |
|  |  | (b) Give reasons for non-participation at each stage  **Section results/ subsection: study population and consultations characteristics, paragraph 1**  **Figure 1** |
|  |  | (c) Consider use of a flow diagram  **Figure 1** |
| Descriptive data | 14* | 1. Give characteristics of study participants (eg demographic, clinical, social) and information on exposures and potential confounders  **Section results, subsection: Study population and consultations characteristics** **S5 Table** |
|  |  | (b) Indicate number of participants with missing data for each variable of interest  **Table 1 and S5 Table** |
|  |  | (c) Summarise follow-up time (eg, average and total amount):  **Section results, subsection: study population and consultations characteristics, paragraph 1** |
| Outcome data | 15* | Report numbers of outcome events or summary measures over time  Section results, subsection: inappropriate antibiotic prescription  **Figure 2** |
| Main results | 16 | (*a*) Give unadjusted estimates and, if applicable, confounder-adjusted estimates and their precision (eg, 95% confidence interval). Make clear which confounders were adjusted for and why they were included  **Section results, subsection: determinants of inappropriate antibiotic prescribing**  **Table 2** |
|  |  | *(b)*Report category boundaries when continuous variables were categorized  **Table 2** |
|  |  | (*c*) If relevant, consider translating estimates of relative risk into absolute risk for a meaningful time period **NA** |
| Other analyses | 17 | Report other analyses done—eg analyses of subgroups and interactions, and sensitivity analyses  **Section results, subsection: determinants of inappropriate antibiotic prescribing, paragraph 5**  **S4Table** |
| **Discussion** | | |
| Key results | 18 | Summarise key results with reference to study objectives  **Section discussion, paragraph 1** |
| Limitations | 19 | Discuss limitations of the study, taking into account sources of potential bias or imprecision. Discuss both direction and magnitude of any potential bias  **Section discussion, subsection: paragraph 14-16** |
| Interpretation | 20 | Give a cautious overall interpretation of results considering objectives, limitations, multiplicity of analyses, results from similar studies, and other relevant evidence  **Section discussion, subsection: paragraphs 2-11** |
| Generalisability | 21 | Discuss the generalisability (external validity) of the study results  **Section discussion, subsection: paragraph 14-16** |
| **Other information** | | |
| Funding | 22 | Give the source of funding and the role of the funders for the present study and, if applicable, for the original study on which the present article is based  **Reported with submission** |
