# Supplementary material for: Inappropriate antibiotic prescribing and its determinants among outpatient children in 3 low- and middle-income countries: A multicentric community-based cohort study
Source: PLoS Med. 2023 Jun 6;20(6):e1004211. doi: 10.1371/journal.pmed.1004211 (PMC10243627; doi:10.1371/journal.pmed.1004211)
Supplement: S1 Table — (DOCX) [file pmed.1004211.s002.docx]

**S1 Table**: List of diagnoses defined as "not requiring antibiotic therapy”, including

diagnoses classified by the two-step algorithm to determine which health events do not require antibiotics in the absence of discriminating criteria.

Step 1: List of diagnoses defined as "not requiring antibiotic therapy”

Step 2: List of diagnoses defined as "not requiring antibiotic therapy” in the absence of discriminating criteria

| **Diagnosis** | **Discriminating criteria** | **Diagnosis** | **Discriminating criteria** |
| --- | --- | --- | --- |
| **Step 1** |  |  |  |
| Allergy | None | Intestinal parasite | None |
| Anemia | None | Jaundice | None |
| Asthma | None | Malaria | None |
| Cardiopathy | None | Congenital malformation | None |
| Conjunctivitis | None | Mumps | None |
| Convulsion | None | Mycosis | None |
| Delivery complication | None | No diagnosis | None |
| Edema | None | Non-infectious disease | None |
| Epilepsy | None | Rhinopharyngitis | None |
| Fetal Alcohol syndrome | None | Rubella | None |
| Good health | None | Skin allergy problem | None |
| Hepatitis | None | Stunted growth | None |
| Hernia (inguinal or umbilical) | None | Teething | None |
| Hypocalcemia | None | Tumor nodule | None |
| Hypothermia | None | Varicella | None |
| Indigestion | None | Viral infection | None |
| Infant colic | None | Wound | None |
| Infant discomfort | None |  |  |
| **Step 2** |  |  |  |
| Lower respiratory infection | Severity score IMCI* >0 or chronic cough (>4 weeks) | Isolated fever | Age <8 weeks |
| Bronchiolitis | high persisting fever or associated pneumonia at chest x-ray or associated otitis media | Laryngotracheitis | Severity score IMCI >0 |
| Gastroenteritis | Bloody stool | Skin infection | Extensive impetigo or dermohypodermitis/sucutaneous abscess or severe furonculosis |
| Isolated fever | Severity score IMCI = 2 & age ≥ 8 weeks | Measles | Severity score IMCI = 2 |
|  |  |  |  |

* IMCI, Integrated Management of Childhood Illness
