# Supplementary material for: Inappropriate antibiotic prescribing and its determinants among outpatient children in 3 low- and middle-income countries: A multicentric community-based cohort study
Source: PLoS Med. 2023 Jun 6;20(6):e1004211. doi: 10.1371/journal.pmed.1004211 (PMC10243627; doi:10.1371/journal.pmed.1004211)
Supplement: S2 Table — (DOCX) [file pmed.1004211.s003.docx]

**S2 Table:** Analysis of the determinants of antibiotic prescription among consultations of children of the 3 countries (Cambodia, Madagascar, Senegal) with a diagnosis not requiring antibiotic therapy without any covariate: country interaction terms.

|  | | **Madagascar, Cambodia and Senegal**  n=10,336 consultations among 2,509 children* | |
| --- | --- | --- | --- |
| **Variables** | | **Adjusted OR (95% CI)** | **P^1^** |
| **Age** | <3mo | ref | **<0.001** |
| 3mo – 1yr | | **2.32** (2.01-2.68) |  |
| >1yr | | **3.73** (3.14-4.34) |  |
| **Weight z-score** | Normal | ref | 0.678 |
| Underweight | | 1.04 (0.88-1.23) |  |
| **History of hospitalization in the last 90 days** = Yes | | 0.97 (0.78-1.21) | 0.803 |
| **History of antibiotic prescription in the last 15 days** = Yes | | 1.46 (1.13-1.89) | 0.004 |
| **Severity score** | 0 | ref | **<0.001** |
| 1 | | **2.00** (1.74-2.28) |  |
| 2 | | **3.21** (2.55-4.04) |  |
| **Season =** Rainy | | **1.33** (1.19-1.48) | **<0.001** |
| **Complicated delivery** = Yes | | 1.06 (0.82-1.36) | 0.660 |
| **Country** | Cambodia | ref | **<0.001** |
| Madagascar | | **0.12** (0.11-0.14) |  |
| Senegal | | **1.95** (1.48-2.58) |  |
| **Site** = Rural | | **2.31** (2.03-2.63) | **<0.001** |
| **Sex** = Male | | 1.09 (0.97-1.22) | 0.132 |
| **Mother’s level of education** | |  |  |
| None or primary school | | ref | 0.210 |
| Incomplete secondary | | 0.89 (0.77-1.02) |  |
| Secondary or university | | 0.90 (0.75-1.08) |  |
| **Mother’s age** | <26yrs | 1.10 (0.96-1.26) | 0.157 |
| **Mother’s profession** | Manual | ref | 0.285 |
| Executive or office job | | 0.95 (0.71-1.27) |  |
| Student or unemployed | | 0.90 (0.79-1.03) |  |
| **Parity** | First child | 0.97 (0.84-1.11) | 0.626 |
| **History of deceased child** | Yes | 0.99 (0.78-1.25) | 0.930 |
| **House density** | Normal | ref | 0.008 |
| Overcrowded (4 or more) | | 1.18 (1.04-1.33) |  |
| **Place of delivery** | Health facility | ref | 0.285 |
| At home | | 1.08 (0.94-1.26) |  |
| 1 P-values are calculated from individual Wald tests  Ref, reference; OR, odds ratio; 95% CI, 95% confidence interval | | | |
