# Supplementary material for: Inappropriate antibiotic prescribing and its determinants among outpatient children in 3 low- and middle-income countries: A multicentric community-based cohort study
Source: PLoS Med. 2023 Jun 6;20(6):e1004211. doi: 10.1371/journal.pmed.1004211 (PMC10243627; doi:10.1371/journal.pmed.1004211)
Supplement: S3 Table — (DOCX) [file pmed.1004211.s004.docx]

**S3 Table**: Results (χ2, degrees of freedom, p-values) of including covariate:country interaction terms in the regression model, where each interaction is considered one at a time.

| Interaction tested | χ ² | Degrees of freedom | P |
| --- | --- | --- | --- |
| **Age** | **28.49** | **3** | **<0.001** |
| **Weight z-score** | 6.48 | 2 | 0.039 |
| **History of hospitalization in the last 90 days** | 0.15 | 1 | 0.702 |
| **History of antibiotic prescription in the last 15 days** | 0.58 | 2 | 0.744 |
| **Severity score** | 5.48 | 4 | 0.241 |
| **Season** | 0.03 | 2 | 0.986 |
| **Complicated delivery** | 8.69 | 2 | 0.013 |
| **Site** | **25.94** | **2** | **<0.001** |
| **Sex** | 9.93 | 2 | 0.007 |
| **Mother’s level of education** | 10.17 | 4 | 0.037 |
| **Mother’s age** | 1.01 | 2 | 0.602 |
| **Mother’s profession** | 13.21 | 4 | 0.010 |
| **Parity** | 1.60 | 2 | 0.449 |
| **History of deceased child** | 1.27 | 2 | 0.528 |
| **House density** | 2.12 | 2 | 0.345 |
| **Place of delivery** | 0.27 | 2 | 0.873 |
