# Supplementary material for: Inappropriate antibiotic prescribing and its determinants among outpatient children in 3 low- and middle-income countries: A multicentric community-based cohort study
Source: PLoS Med. 2023 Jun 6;20(6):e1004211. doi: 10.1371/journal.pmed.1004211 (PMC10243627; doi:10.1371/journal.pmed.1004211)
Supplement: S4 Table — (DOCX) [file pmed.1004211.s005.docx]

**S4 Table** Analysis of the determinants of antibiotic prescription among consultations with a diagnosis not requiring antibiotic therapy, with a separate model considered for each country.

|  | | | **Madagascar** n=7,960 consultations among 1,732 children* | | | **Cambodia** n=2,051 consultations among 540 children* | | | **Senegal** n=388 consultations among 272 children* | | |
| --- | --- | --- | --- | --- | --- | --- | --- | --- | --- | --- | --- |
| **Variables** | | | **OR (95% CI)** | **p^1^** | **Adjusted OR (95% CI)** | **OR (95% CI)** | **p** | **Adjusted OR (95% CI)** | **OR (95% CI)** | **p** | **Adjusted OR (95% CI)** |
| **Age** | <3mo | | ref | **<0.001** | ref | ref | **<0.001** | ref | ref | **<0.001** | ref |
| 3mo – 1yr | | | **1.74** (1.51-2.05) |  | **1.91** (1.63-2.23) | **3.52** (2.66-4.67) |  | **4.12** (3.08-5.10) | **2.50** (1.55-4.05) |  | **3.02** (1.77-5.16) |
| >1yr | | | **3.46** (2.83-4.23) |  | **3.65** (2.98-4.48) | **3.80** (2.84-5.10) |  | **4.80** (3.55-6.48) | **N/A** |  | **N/A** |
| **Weight z-score** | Normal | | ref | 0.121 |  | ref | 0.317 | ref | ref | 0.561 |  |
| Underweight | | | 1.17 (0.95-1.45) |  |  | 0.85 (0.63-1.16) |  | 0.80 (0.59-1.08) | 1.22 (0.62-2.38) |  |  |
| **History of hospitalization in the last 90 days** = Yes | | | **0.71** (0.51-0.99) | **0.05** |  | 1.00 (0.76-1.32) | 0.987 |  | N/A |  |  |
| **History of antibiotic prescription in the last 15 days** = Yes | | | **1.43** (1.01-2.02) | **0.049** | 1.40 (0.98-1.98) | **1.28** (1.01-2.18) | **0.043** | **1.69** (1.14-2.50) | 0.75 (0.17-3.28) | 0.700 |  |
| **Severity score** | 0 | | ref | **<0.001** | ref | ref | **<0.001** | ref | ref | **0.009** | ref |
| 1 | | | **2.60** (2.20-3.07) |  | **1.99** (1.68-2.36) | **2.25** (1.75-2.88) |  | **1.98** (1.54-2.56) | 2.34 (1.31-4.20) |  | **2.26** (1.19-4.26) |
| 2 | | | **2.46** (1.89-3.21) |  | **3.41** (2.58-4.51) | **2.20** (1.49-3.25) |  | **2.93** (1.91-4.52) | 1.86 (0.83-4.14) |  | **1.92** (0.81-4.50) |
| **Season =** Rainy | | | **1.42** (1.25-1.62) | **<0.001** | **1.34** (1.18-1.53) | **1.40** (1.16-1.69) | **<0.001** | **1.26** (1.04-1.53) | **1.78** (1.12-2.82) | **0.014** | **1.64** (1.02-2.62) |
| **Complicated delivery** = Yes | | | **0.65** (0.51-0.83) | **<0.001** |  | **0.32** (0.21-0.51) | **<0.001** |  | 0.79 (0.35-1.75) | 0.556 |  |
| **Site** = Rural | | | **1.94** (1.67-2.25) | **<0.001** | **1.82** (1.56-2.14) | **3.96** (3.10-5.07) | **<0.001** | **4.00** (3.12-5.13) | 2.52 (1.35-4.68) | **0.003** | **4.15** (2.15-8.00) |
| **Sex** = Male | | | **1.25** (1.07-1.45) | **<0.001** | **1.22** (1.06-1.41) | 0.88 (0.70-1.11) | 0.287 | 0.82 (0.68-1.00) | 1.04 (0.67-1.62) | 0.859 | 1.04 (0.65-1.64) |
| **Mother’s level of education** | | |  |  |  |  |  |  |  |  |  |
| None or primary school | | | ref | **<0.001** | ref | ref | **<0.001** |  | ref | 0.485 |  |
| Incomplete secondary | | | **0.79** (0.66-0.94) |  | **0.82** (0.69-0.97) | 0.92 (0.73-1.18) |  |  | 1.43 (0.76-2.71) |  |  |
| Secondary or university | | | **0.63** (0.51-0.78) |  | **0.74** (0.60-0.91) | 1.43 (1.01-2.02) |  |  | 1.29 (0.55-3.07) |  |  |
| **Mother’s age** | <26yrs | | 1.01 (0.87-1.17) | 0.890 |  | 1.05 (0.83-1.32) | 0.653 |  | 1.35 (0.85-2.16) | 0.209 |  |
| **Mother’s profession** | Manual | | ref | 0.057 |  | ref | **<0.001** |  | ref | 0.239 |  |
| Executive or office job | | | 0.75 (0.53-1.05) |  |  | **4.40** (2.02-9.64) |  |  | 2.75 (0.45-16.96) |  |  |
| Student or unemployed | | | 1.09 (0.91-1.30) |  |  | **0.75** (0.60-0.94) |  |  | 1.50 (0.89-2.54) |  |  |
| **Parity** | First child | | **0.85** (0.73-0.99) | **0.042** |  | 0.99 (0.79-1.25) | 0.978 |  | 1.50 (0.89-2.54) | 0.129 |  |
| **History of deceased child** | | Yes | 1.06 (0.8-1.4) | 0.642 |  | 0.85 (0.46-1.58) | 0.618 |  | 0.92 (0.34-2.52) | 0.870 |  |
| **House density** | Normal | | ref | 0.086 | ref | ref | 0.911 |  | ref | 0.545 |  |
| Overcrowded (4 or more) | | | 1.14 (0.97-1.34) |  | **1.16** (1.00-1.36) | 1.01 (0.80-1.27) |  |  | 1.16 (0.72-1.88) |  |  |
| **Place of delivery** | Health facility | | ref | **<0.001** |  | ref | 0.509 |  | ref | 0.127 |  |
| At home | | | **1.36** (1.17-1.59) |  | 0.88 (0.76-1.03) | 1.45 (0.48-4.33) |  |  | 0.42 (0.14-1.28) |  |  |
| 1 P-values are calculated from individual Wald tests  * Analysis performed on consultations with complete dataset (for details, see S5 Table)  The model for each country is adjusted for the variables listed in the "adjusted OR” column of each country. Confidence intervals for the adjusted ORs should be interpreted with caution: their ranges may be underestimated due to the fact that the variable selection --- through minimisation of the AIC --- and the estimation of adjusted ORs were carried out on the same dataset.  In Senegal, ORs associated with age at consultation>3 months should be interpreted only for infants residing in the urban site (no infants >3 months were included in the rural site)  Ref, reference; OR, odds ratio; 95% CI, 95% confidence interval; N/A, not available. | | | | | | | | | | | |
