# Supplementary material for: Inappropriate antibiotic prescribing and its determinants among outpatient children in 3 low- and middle-income countries: A multicentric community-based cohort study
Source: PLoS Med. 2023 Jun 6;20(6):e1004211. doi: 10.1371/journal.pmed.1004211 (PMC10243627; doi:10.1371/journal.pmed.1004211)
Supplement: S5 Table — (DOCX) [file pmed.1004211.s006.docx]

**S5 TABLE:** Characteristics of consultations determined not to require antibiotic therapy.

|  | | **Cambodia**  2,060 consultations among 541 children | | **Madagascar**  7,961 consultations among 1,733 children | | **Senegal**  395 consultations among 277 children | |
| --- | --- | --- | --- | --- | --- | --- | --- |
| **Variables** | | **No Antibiotic**  **n (%)** | **Receiving antibiotic**  **n (%)** | **No Antibiotic**  **n (%)** | **Receiving antibiotic**  **n (%)** | **No Antibiotic n (%)** | **Receiving antibiotic**  **n (%)** |
| **Consultations** | | **886** | **1,174** | **6,722** | **1,239** | **169** | **226** |
| **Age** | <3mo | 247 (27.9) | 126 (10.7) | 2779 (41.3) | 330 (26.6) | 131 (77.5) | 133 (58.8) |
| 3mo – 1yr | | 288 (32.5) | 486 (41.4) | 3228 (48.0) | 646 (52.1) | 38 (22.5) | 93 (40.7) |
| >1yr | | 351 (39.6) | 562 (47.9) | 715 (10.6) | 263 (21.2) | - | - |
| **Weight z-score** | Normal | 768 (86.7) | 1040 (88.6) | 5960 (88.7) | 1078 (87.0) | 145 (85.8) | 196 (86.7) |
| Underweight | | 114 (12.9) | 129 (11.0) | 758 (11.3) | 160 (12.9) | 18 (10.7) | 29 (12.8) |
| *Missing data* | | 4 (0.5) | 5 (0.4) | 4 (0.1) | 1 (0.1) | 6 (3.6) | 1 (0.4) |
| **History of hospitalization in the last 90 days** | | 121 (13.7) | 162 (13.8) | 351 (5.2) | 47 (3.8) | - | - |
| **History of antibiotic prescription in the last 15 days** | | 47 (5.3) | 97 (8.3) | 162 (2.4) | 50 (4.0) | 4 (2.4) | 5 (2.2) |
| **Severity score** | 0 | 719 (81.2) | 778 (66.3) | 5740 (85.4) | 864 (69.7) | 137 (81.1) | 151 (66.8) |
| 1 | | 122 (13.8) | 290 (24.7) | 719 (10.7) | 283 (22.8) | 21 (12.4) | 53 (23.5) |
| 2 | | 45 (5.1) | 106 (9.0) | 263 (3.9) | 92 (7.4) | 11 (6.5) | 22 (9.7) |
| **Season (**Rainy) | | 385 (43.5) | 609 (51.9) | 3443 (51.2) | 736 (59.4) | 60 (35.5) | 108 (47.8) |
| **Complicated delivery** | | 70 (7.9) | 29 (2.5) | 619 (9.2) | 79 (6.4) | 14 (8.3) | 15 (6.6) |
| *Missing data* | | 2 (0.2) | 6 (0.5) | _ | _ | 18 (10.7) | 23 (10.2) |
| **Children** | | **257** | **284** | **1521** | **212** | **117** | **160** |
| **Site** (Rural) | | 67 (26.1) | 172 (60.6) | 922 (60.6) | 136 (64.2) | 16 (13.7) | 55 (34.4) |
| **Sex** (Male) | | 134 (52.1) | 129 (45.4) | 776 (51.0) | 115 (54.2) | 61 (52.1) | 79 (49.4) |
| *Missing data* | | 1 (0.4) | 0 (0.0) | - | - | 4 (3.4) | 1 (0.6) |
| **Mother’s level of education** | |  |  |  |  |  |  |
| None or primary school | | 146 (56.8) | 149 (52.5) | 355 (23.3) | 58 (27.4) | 94 (80.3) | 115 (71.9) |
| Incomplete secondary | | 81 (31.5) | 90 (31.7) | 787 (51.7) | 110 (51.9) | 15 (12.8) | 29 (18.1) |
| Secondary or university | | 30 (11.7) | 45 (15.8) | 379 (24.9) | 44 (20.8) | 8 (6.8) | 16 (10.0) |
| **Mother’s age** = <26yrs | | 110 (42.8) | 123 (43.3) | 846 (55.6) | 124 (58.5) | 36 (30.8) | 61 (38.1) |
| **Mother’s profession** | |  |  |  |  |  |  |
| Manual | | 139 (54.1) | 169 (59.5) | 371 (24.4) | 52 (24.5) | 32 (27.4) | 32 (20.0) |
| Executor of office job | | 5 (1.9) | 18 (6.3) | 86 (5.7) | 12 (5.7) | 1 (0.9) | 5 (3.1) |
| Student or unemployed | | 113 (44.0) | 97 (34.2) | 1063 (69.9) | 148 (69.8) | 84 (71.8) | 123 (76.9) |
| *Missing data* | | - | - | 1 (0.1) | 0 (0.0) | - | - |
| **Primiparity** | | 107 (41.6) | 121 (42.6) | 547 (36.0) | 95 (44.8) | 24 (20.5) | 41 (25.6) |
| *Missing data* | | - | - | 1 (0.1) | 0 (0.0) | - | - |
| **History of deceased child** | | 9 (3.5) | 8 (2.8) | 114 (7.5) | 13 (6.1) | 7 (6.0) | 7 (4.4) |
| *Missing data* | | - | - | 1 (0.1) | 0 (0.0) | - | - |
| **House density**  (Overcrowded, 4 or more) | | 111 (43.2) | 144 (50.7) | 521 (34.3) | 72 (34.0) | 35 (29.9) | 55 (34.4) |
| *Missing data* | | 1 (0.4) | 0 (0.0) | 1 (0.1) | 0 (0.0) | 1 (0.9) | 4 (2.5) |
| **Place of delivery** | |  |  |  |  |  |  |
| Health facility | | 254 (98.8) | 278 (97.9) | 959 (63.1) | 120 (56.6) | 109 (93.2) | 154 (96.2) |
| At home | | 2 (0.8) | 6 (2.1) | 562 (36.9) | 92 (0.43) | 7 (6.0) | 6 (3.8) |
| *Missing data* | | 1 (0.4) | 0 (0.0) | - | - | 1 (0.9) | 0 (0.0) |
