# Supplementary material for: Inappropriate antibiotic prescribing and its determinants among outpatient children in 3 low- and middle-income countries: A multicentric community-based cohort study
Source: PLoS Med. 2023 Jun 6;20(6):e1004211. doi: 10.1371/journal.pmed.1004211 (PMC10243627; doi:10.1371/journal.pmed.1004211)
Supplement: S6 Table — N = 4,648 antibiotic prescriptions across all countries. (DOCX) [file pmed.1004211.s007.docx]

**S6 Table:** Distribution of antibiotics prescribed across all consultations, stratified by country. N= 4,648 antibiotic prescriptions across all countries.

| **CAMBODIA N = 1829** | | | **MADAGASCAR N = 2488** | | | **SENEGAL N = 331** | | |
| --- | --- | --- | --- | --- | --- | --- | --- | --- |
| Antibiotic name | n | % | Antibiotic name | n | % | Antibiotic name | n | % |
| Amoxicillin | 653 | 35.7% | Amoxicillin | 597 | 24.0% | Cefixime | 114 | 34.4% |
| Ampicillin | 276 | 15.1% | Gentamicin | 439 | 17.6% | Amoxicillin/  Clavulanic Acid | 58 | 17.5% |
| Gentamicin | 199 | 10.9% | Amoxicillin/  Clavulanic Acid | 419 | 16.8% | Amoxicillin | 49 | 14.8% |
| Ceftriaxone | 97 | 5.3% | Ceftriaxone | 318 | 12.8% | Ceftriaxone | 30 | 9.1% |
| Erythromycin | 91 | 5.0% | Cefixime | 162 | 6.5% | Gentamicin | 12 | 3.6% |
| Sulfamethoxazole/  trimethoprim | 81 | 4.4% | Ampicillin | 137 | 5.5% | Other | 11 | 3.3% |
| Amoxicillin/  Clavulanic Acid | 70 | 3.8% | Metronidazole | 96 | 3.9% | Erythromycin | 11 | 3.3% |
| Cefixime | 64 | 3.5% | Erythromycin | 95 | 3.8% | Cefotaxime | 8 | 2.4% |
| Metronidazole | 63 | 3.4% | Others | 34 | 1.4% | Cephalosporine | 7 | 2.1% |
| Tobramycin | 49 | 2.7% | Ciprofloxacin | 30 | 1.2% | Josamycin | 6 | 1.8% |
| Cefdinir | 37 | 2.0% | Neomycin | 28 | 1.1% | Cefadroxil | 5 | 1.5% |
| Ciprofloxacin | 36 | 2.0% | Oxacillin | 27 | 1.1% | Cefpodoxime | 5 | 1.5% |
| Cefalexin | 23 | 1.3% | Sulfamethoxazole/  trimethoprim | 26 | 1.0% | Ornidazole | 4 | 1.2% |
| Cloxacillin | 22 | 1.2% | Fusidic Acid | 12 | 0.5% | Cloxacillin | 3 | 0.9% |
| Others | 20 | 1.1% | Cefpodoxime | 12 | 0.5% | Azithromycin | 2 | 0.6% |
| Fusidic Acid | 18 | 1.0% | Josamycin | 8 | 0.3% | Cephalexin | 1 | 0.3% |
| Nifuroxazide | 8 | 0.4% | Cephalexin | 6 | 0.2% | Cefatrizine | 1 | 0.3% |
| Azithromycin | 4 | 0.2% | Ofloxacin | 6 | 0.2% | Ciprofloxacin | 1 | 0.3% |
| Ofloxacin | 4 | 0.2% | Cefotaxime | 5 | 0.2% | Metronidazole | 1 | 0.3% |
| Cephalosporin | 3 | 0.2% | Cefadroxil | 4 | 0.2% | Ofloxacin | 1 | 0.3% |
| Neomycin | 3 | 0.2% | Thiamphenicol | 4 | 0.2% | Oxacillin | 1 | 0.3% |
| Penicillin V | 3 | 0.2% | Chloramphenicol | 3 | 0.1% |  |  |  |
| Moxifloxacin | 2 | 0.1% | Nifuroxazide | 3 | 0.1% |  |  |  |
| Cefaclor | 1 | 0.1% | Penicillin G | 3 | 0.1% |  |  |  |
| Norfloxacin | 1 | 0.1% | Amikacin | 2 | 0.1% |  |  |  |
| Sulfadiazine/  trimethoprim | 1 | 0.1% | Cefuroxime | 2 | 0.1% |  |  |  |
|  |  |  | Imipenem/cilastatin | 2 | 0.1% |  |  |  |
|  |  |  | Cefepime | 1 | 0.0% |  |  |  |
|  |  |  | Cloxacillin | 1 | 0.0% |  |  |  |
|  |  |  | Ertapenem | 1 | 0.0% |  |  |  |
|  |  |  | Fosfomycin | 1 | 0.0% |  |  |  |
|  |  |  | Penicillin V | 1 | 0.0% |  |  |  |
|  |  |  | Colistin | 1 | 0.0% |  |  |  |
|  |  |  | Sulfadimidine/  trimethoprim | 1 | 0.0% |  |  |  |
|  |  |  | Sulfamerazine/  trimethoprim | 1 | 0.0% |  |  |  |
